# Rat DCST1 and DCST2 are essential for sperm–egg fusion^†^

**DOI:** 10.1093/biolre/ioag111

**Published:** 2026-05-23

**Authors:** Motochika Nakano, Katsuma Yamaga, Reika Uriu, Ayumu Taira, Keisuke Masuda, Kimi Araki, Ayako Isotani, Shunsuke Yuri, Toru Takeo, Masahito Ikawa, Taichi Noda

**Affiliations:** Division of Reproductive Biology, Institute of Resource Development and Analysis, Kumamoto University, Kumamoto, Japan; Division of Developmental Genetics, Institute of Resource Development and Analysis, Kumamoto University, Kumamoto, Japan; Division of Reproductive Engineering, Institute of Resource Development and Analysis, Kumamoto University, Kumamoto, Japan; Division of Reproductive Biology, Institute of Resource Development and Analysis, Kumamoto University, Kumamoto, Japan; Division of Developmental Genetics, Institute of Resource Development and Analysis, Kumamoto University, Kumamoto, Japan; Division of Reproductive Biology, Institute of Resource Development and Analysis, Kumamoto University, Kumamoto, Japan; Division of Developmental Genetics, Institute of Resource Development and Analysis, Kumamoto University, Kumamoto, Japan; Division of Reproductive Engineering, Institute of Resource Development and Analysis, Kumamoto University, Kumamoto, Japan; Division of Developmental Genetics, Institute of Resource Development and Analysis, Kumamoto University, Kumamoto, Japan; Center for Metabolic Regulation of Healthy Aging, Kumamoto University, Kumamoto, Japan; Life Science Collaboration Center (LiSCo), Nara Institute of Science and Technology, Nara, Japan; Life Science Collaboration Center (LiSCo), Nara Institute of Science and Technology, Nara, Japan; Laboratory of Experimental Animals, Research Institution, National Center for Geriatrics and Gerontology, Obu, Japan; Division of Reproductive Engineering, Institute of Resource Development and Analysis, Kumamoto University, Kumamoto, Japan; Department of Experimental Genome Research, Research Institute for Microbial Diseases, The University of Osaka, Osaka, Japan; Division of Reproductive Biology, Institute of Resource Development and Analysis, Kumamoto University, Kumamoto, Japan

**Keywords:** fertilization, genetically modified rats, infertility, IZUMO1

## Abstract

Physiological functions of fertilization-related genes in male fertility differ among species. The sperm membrane proteins, DC-STAMP domain containing (DCST) 1 and 2, are essential for male fertility in mice and zebrafish. Although *dcst2* knockout (KO) zebrafish sperm showed impaired binding to the oolemma, mouse *Dcst2* KO sperm showed impaired fusion after binding to the oolemma. Thus, the physiological functions of the target genes must be analyzed in various genetically modified animals. In this study, the physiological function of DCST1/2 in male fertility was examined in *Dcst1/2* double-KO (dKO) rats. *Dcst1/2* dKO male rats were completely infertile in mating tests, which was consistent with the phenotypes observed in *Dcst1/2* dKO mice and zebrafish. The motility and acrosome reaction of *Dcst1/2* dKO rat sperm were similar to those of control sperm. *Dcst1/2* dKO sperm accumulated in the perivitelline space of eggs collected from females after natural mating, similar to izumo sperm–egg fusion 1 (*Izumo1*) KO rat sperm. Sperm lacking either *Dcst1/2* or *Izumo1* were incubated with zona pellucida-free eggs in vitro. *Izumo1* KO sperm showed impaired sperm–egg binding. IZUMO1 remained in *Dcst1/2* dKO sperm, and *Dcst1/2* dKO sperm after the acrosome reaction could bind to, but not fuse with, the oolemma. Rat DCST1/2 is essential for sperm–egg fusion, which is consistent with the function of mouse DCST1/2. Phylogenetic analyses showed that human DCST1/2 was closer to mouse and rat DCST1/2 than to zebrafish Dcst1/2. The role of DCST1/2 in sperm–egg fusion is therefore widely conserved in mammals.

## Graphical Abstract

**Graphical Abstract:**
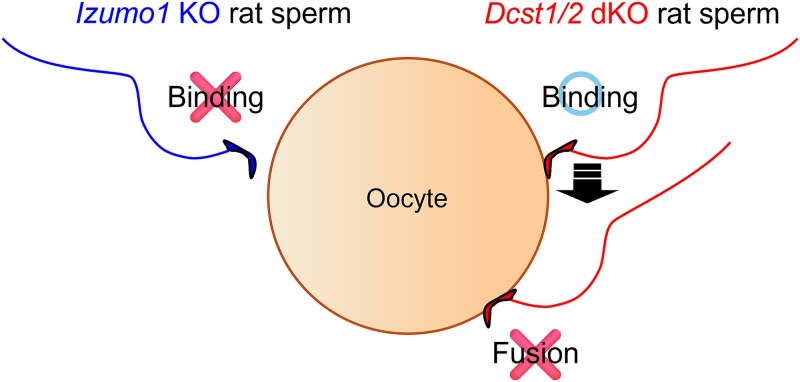

## Teaser

Rat DCST1/2 is necessary for sperm–egg fusion, corresponding to the function of mouse DCST1/2 but not that of zebrafish Dcst1/2.

## Introduction

Acrosome-reacted sperm can bind to and fuse with eggs after passing through the zona pellucida (ZP). Izumo sperm–egg fusion 1 (IZUMO1), a sperm membrane protein, was first identified as a fusion-related sperm factor because *Izumo1* knockout (KO) mouse sperm can bind to, but not fuse with, the oocyte membrane (oolemma) when incubated with ZP–free eggs [1]. JUNO (also known as IZUMO1r or FOLR4) was identified as a receptor for sperm IZUMO1 by biophysical analysis [2, 3]. However, *Izumo1*-expressing cultured cells incubated with ZP–free eggs can bind to, but not fuse with, these eggs [4]. The binding and fusion abilities of mouse *Izumo1* KO sperm with eggs were re-evaluated using fluorescently labeled sperm with green fluorescent protein (GFP) in the acrosome and DsRed in the middle piece; *Izumo1* KO sperm bound to the oolemma were acrosome-intact [5]. Under natural conditions, only acrosome-reacted sperm can interact with the oolemma. Rat *Izumo1* KO sperm rarely bound to the oolemma [5]. Therefore, the IZUMO1–JUNO interaction is essential for sperm–egg binding.

Genome editing accelerates the phenotypic screening of target genes at an individual level. Phenotypic screening of genetically modified mice and zebrafish lacking testis-enriched genes indicated the sperm–egg fusion-related proteins: RIKEN cDNA 4930451I11 (also known as FIMP) [6], LLLL and CFNLAS motif containing 1 (LLCFC1) (also known as SOF1), transmembrane protein (TMEM) 95, sperm acrosome associated 6 (SPACA6) [4, 7, 8], DC-STAMP domain containing (DCST) 1, DCST2 [9, 10], and TMEM81 [11]. For example, DCST1 and DCST2 are multi-pass transmembrane proteins abundant in the testes. They conserve a dendritic cell-specific transmembrane protein (DC-STAMP)-like domain. *Dcstamp* and osteoclast stimulatory transmembrane protein (*Ocstamp*) are paralog genes of mouse *Dcst1/2*. These genes are indispensable for cell–cell fusion in osteoclasts and foreign body giant cells [12–14], indicating that proteins containing DC-STAMP-like domains have a critical role in cell–cell fusion. The disruption of *Dcst1/2* causes male infertility in mice and zebrafish, but the phenotypes of male infertility differ slightly between these species. Zebrafish *dcst2* KO sperm cannot bind to the oolemma, whereas mouse *Dcst2* KO sperm can bind to but not fuse with the oolemma [10]. This result is consistent with the difference in physiological functions between mouse and zebrafish *Tmem81* mutants in sperm–egg interactions [11]. Acrosin, a serine protease in the acrosomal region that supports sperm penetration into the ZP, is required for male fertility in rats and hamsters, whereas mouse acrosin is dispensable for sperm fertilization ability [15–17]. These results highlight the importance of analyzing target genes in various genetically modified animals to elucidate the fertilization mechanisms in mammals. In the present study, the physiological functions of rat DCST1/2 in male fertility were analyzed by establishing genetically modified rat models.

## Materials and methods

### Samples

Rats were purchased from Japan SLC (Shizuoka, Japan) and the Institute for Animal Reproduction (Ibaraki, Japan). The rats were acclimated to a 12-h light/12-h dark cycle with ad libitum access to food and water. All animal experiments were approved by the Animal Care and Use Committee of Kumamoto University (approval numbers A2023-021 and A2025-012). Frozen sperm from mutant rats established in this study will be deposited at the Center for Animal Resources and Development Rat Bank (Kumamoto University) [18]. *Izumo1* KO rats generated in a previous study were used in this study [5].

### Generation of *Dcst1/2* mutant rats

Pregnant mare serum gonadotropin (PMSG) (20–30 units; ASKA Animal Health, Tokyo, Japan) was injected into the abdominal cavity of Iar:Wistar–Imamichi female rats, followed by the injection of human chorionic gonadotropin (hCG) (20–30 units; ASKA Animal Health). Fifteen hours after the hCG injection, eggs were collected from the ampulla. Cauda epididymal sperm from Iar:Wistar–Imamichi male rats were pre-incubated in modified human tubal fluid (mHTF) medium [19] for 1–2 h, and then inseminated into eggs. Six hours after insemination, the cumulus cells of the eggs were removed using hyaluronidase (Sigma-Aldrich, St. Louis, MO, USA) at a final concentration of 1 mg/mL. A mixture of guide RNAs (gRNAs) [5′-CAGCTCATACACCATCTGTG-3′ (gRNA #1) and 5′-TTGACAACGTCTATATCACC-3′ (gRNA #2)] (Sigma-Aldrich) and Cas9 enzyme (Thermo Fisher Scientific, Waltham, MA, USA) (final concentrations: Cas9, 27 ng/μL; gRNA #1, 20 ng/μL; and gRNA #2, 20 ng/μL) was injected into a pronucleus. The injected eggs were then transferred to the ampulla of pseudo-pregnant female rats mated with vasectomized male rats. Offspring were obtained by natural birth 22–23 days after embryo transfer. The genotype was identified using polymerase chain reaction (PCR) with KOD One (TOYOBO, Osaka, Japan) and the following primers [wild-type (WT) allele (264 bp): 5′-TCAGAGCCCTCTATTATCGG-3′ (Fw 1) and 5′-CTCTCTCTAGCCCCTTATGC-3′ (Rv 2), and KO allele (463 bp for *em1*, and 456 bp for *em2*): 5′-TGAACTTCATTGGCAGGCAG-3′ (Rv 1), and 5′-CTCTCTCTAGCCCCTTATGC-3′ (Rv 2)]. The PCR conditions were as follows: amplification for 3 min at 98°C; followed by 40 cycles of 98°C for 10 s, 55°C for 5 s, and 72°C for 1 s; and extension for 2 min at 72°C. After the F2 generation, mutants were used for phenotypic analyses.

### Detection of *Dcst1* and *Dcst2* mRNAs in a testis

The cDNA synthesis was conducted as previously described [10]. Specifically, the testes of *Dcst1/2* double hetero (dHet) and double KO (dKO) males were homogenized in TRIzol reagent (Thermo Fisher Scientific), and total RNA was extracted according to the manufacturer’s instructions. Total RNA was reverse-transcribed into cDNA using SuperScript IV First-Strand Synthesis System (Invitrogen, Carlsbad, CA, USA) and deoxyribonuclease (RT Grade) (Nippon Gene, Tokyo, Japan). PCR was conducted with KOD One and the following primers [*Dcst1* (475 bp, accession # ENSRNOG00000020621): 5′-GCAGAAGCAGTCTGCAGTGG-3′ (Fw 2) and 5′-GAAGGAGTACTGCACGAAGG-3′ (Rv 3), *Dcst2* (407 bp, accession # ENSRNOG00000058618): 5′-CATTGCTCGGCCGGTTTTTG-3′ (Fw 3), and 5′-GGAGATGCTGTGGATGCTTC-3′ (Rv 4), and *Actb* (201 bp, accession # NM_031144): 5′-CATCCGTAAAGACCTCTATGCCAAC-3′ (Fw), and 5′-ATCTGCTGGAAGGTGGACAG-3′ (Rv)]. The PCR conditions were as follows: amplification for 3 min at 98°C; followed by 40 cycles of 98°C for 10 s, 55°C for 5 s, 72°C for 1 s; and extension for 2 min at 72°C for *Dcst1*. Amplification for 3 min at 98°C; followed by 40 cycles of 98°C for 10 s, 60°C for 5 s, 72°C for 1 s; and extension for 2 min at 72°C was used for *Dcst2* and *Actb*.

#### Mating tests

*Dcst1/2* dHet and dKO males (8–9 weeks old) were caged with two WT rats for more than 2 months. The number of offspring delivered was recorded.

#### Expression patterns in multiple tissues and male germ cells

The expression patterns in multiple tissues and male germ cells were obtained from rat RNA-sequencing (RNA-seq) data (BioProject accession: PRJNA238328) [20] and rat single-cell RNA-seq data [21].

#### Gross morphology and histology of testes

Morphological and histological analyses were conducted as previously described [22]. The testes were collected from *Dcst1/2* dHet and dKO male rats (15–32 weeks old). After measuring testicular weight, the testes were fixed in Bouin’s fluid (Polysciences, Warrington, PA, USA) at 4°C overnight. The fixed testes were dehydrated using increasing ethanol concentrations and embedded in paraffin (Fujifilm Wako, Osaka, Japan). Next, they were sliced to 5-μm thickness using a microtome (REM-710; Yamato Kohki Industrial, Saitama, Japan). The slides were stained with 1% (vol/vol) periodic acid solution (Nacalai Tesque, Kyoto, Japan), followed by treatment with Schiff’s reagent (Merck, Darmstadt, Germany) for 20 min, and Mayer’s Hematoxylin solution (Muto Pure Chemicals, Tokyo, Japan). After dehydration with ethanol, the slides were mounted using Multi Mount 480 (Matsunami Glass, Osaka, Japan) and observed under microscopes [BX-50 (Evident, Osaka, Japan), and BZ-X810 (Keyence, Osaka, Japan)]. The elongated spermatids at stages VII–VIII of the seminiferous tubules were counted, and their areas were measured using ImageJ (https://imagej.net/ij/).

### Sperm morphology, motility, and acrosome reaction

Cauda epididymal sperm from *Dcst1/2* and *Izumo1* mutant rats (12–35 weeks old) were dispersed in phosphate-buffered saline (PBS) (for sperm morphology and acrosome reaction) and mHTF medium (for sperm motility and acrosome reaction). Sperm motility was measured as previously described [22]. Sperm motility after 3 h of incubation in mHTF medium was recorded for 0.75 s using Axiolab5 (Zeiss, Oberkochen, Germany) equipped with MiniTherm stage warmer (Hamilton Thorne, Beverly, MA, USA), a CM-040 GE CCD camera (JAI A/S, Copenhagen, Denmark), and 100 μm-deep chambers (Leja, Nieuw-Vennep, The Netherlands) at 37°C. Sperm parameters were analyzed using a CEROS II sperm analysis system (Hamilton Thorne). To examine the status of the sperm acrosome, cauda epididymal sperm were dispersed in PBS or incubated in mHTF medium. After 1-h incubation in mHTF medium, some sperm were put in an mHTF drop containing either Ca^2+^ ionophore, A23187 (final concentration 20 μM, Merck), or 1% (vol/vol) DMSO (Sigma-Aldrich) for 2 h. After washing with PBS, the sperm suspended in PBS were smeared on a glass slide and dried on a hotplate. Samples were fixed with methanol for 10 min and washed with PBS. After blocking with 10% (vol/vol) goat serum (Thermo Fisher Scientific) for 1 h, the sperm were incubated with a rabbit polyclonal antibody against human IZUMO1 (1:100) [1] overnight. After washing with PBS containing 0.05% (vol/vol) Tween 20 (PBS–Tween20), the sperm were treated with goat anti-rabbit immunoglobulin (IgG) Alexa Fluor 488 (A32731) (1:200, Thermo Fisher Scientific) and Alexa Fluor 568-conjugated lectin peanut agglutinin (PNA) (1:2000; Invitrogen) for 1 h. After washing with PBS–Tween 20, the sperm were counterstained with Hoechst33342 (1:10,000, Invitrogen), washed with PBS, sealed with Immu-Mount (Thermo Fisher Scientific), and observed under a phase-contrast microscope (BX-50) with fluorescence equipment. The acrosome status using PNA lectin was classified as previously described [23].

### Egg observation after mating

Hormone-treated female rats were caged with *Dcst1/2* WT, dHet, and dKO male rats. Successful mating was confirmed by observing copulatory plugs the following morning. Eggs were collected from the ampulla and treated with 1 mg/mL hyaluronidase to remove the cumulus cells. The collected eggs were incubated in mHTF medium to observe embryonic development.

### Observation of the binding and fusion of sperm and eggs

Cauda epididymal sperm of *Dcst1/2* and *Izumo1* mutant rats (12–35 weeks old) were pre-incubated in mHTF medium for 3–6 h. The cumulus cells and ZP in eggs collected from hormone-treated females were removed by treatment with hyaluronidase (final concentration 1 mg/mL) and collagenase (final concentration 1 mg/mL, C1639; Sigma-Aldrich) as described previously [4]. Pre-incubated sperm were inseminated with ZP-free eggs for more than 4 h. After washing with mHTF drops, a differential interference microscope (IX73, Evident) was used to determine whether the mutant sperm were bound to the oolemma. To observe the acrosomal status of sperm bound to the oolemma, pre-incubated sperm were stained with Alexa Fluor 488-conjugated lectin PNA (1:200) for 1 h, and then inseminated into ZP-free eggs for 1–2 h. After washing with mHTF drops, eggs were counterstained with Hoechst33342 (1:10,000) for 10 min, and observed under a fluorescence microscope (BZ-X810). To observe the status of the sperm nuclei bound to the oolemma, some eggs were fixed with either 0.25% (vol/vol) glutaraldehyde (Wako) or 0.2% PFA (Sigma-Aldrich), stained with Hoechst33342 (1:10,000), washed with mHTF drops, and observed under a fluorescence microscope (BZ-X810). The remaining eggs were incubated in mHTF medium to observe embryonic development.

### Phylogenetic tree

Multiple sequence alignment was conducted using Clustal Omega (https://www.ebi.ac.uk/jdispatcher/msa/clustalo) with amino acid sequences [DCST1: D3ZE12 (rat), Q059Y8 (mouse), Q5T197-1 (human), and A0AB32TA10 (zebrafish); and DCST2: A0A0G2K3F3 (rat), A0A140LIJ0 (mouse), Q5T1A1-1 (human), and A0AB13A7T9 (zebrafish)]. Jalview (https://www.jalview.org/) was used to visualize the obtained alignment data and analyze the phylogenetic tree (neighbor joining method).

### Statistical analysis

All data are shown as the mean ± standard deviation (SD) of at least three independent experiments. Significant differences were examined using the Mann–Whitney test (Figures 2D, 2F, and  3B) and the Kruskal–Wallis test (Figure 4B and Supplementary Figure S2) using Prism9 (MDF, Japan).

## Results and discussion

### *Dcst1/2* dKO male rats were infertile

**Figure 1:**
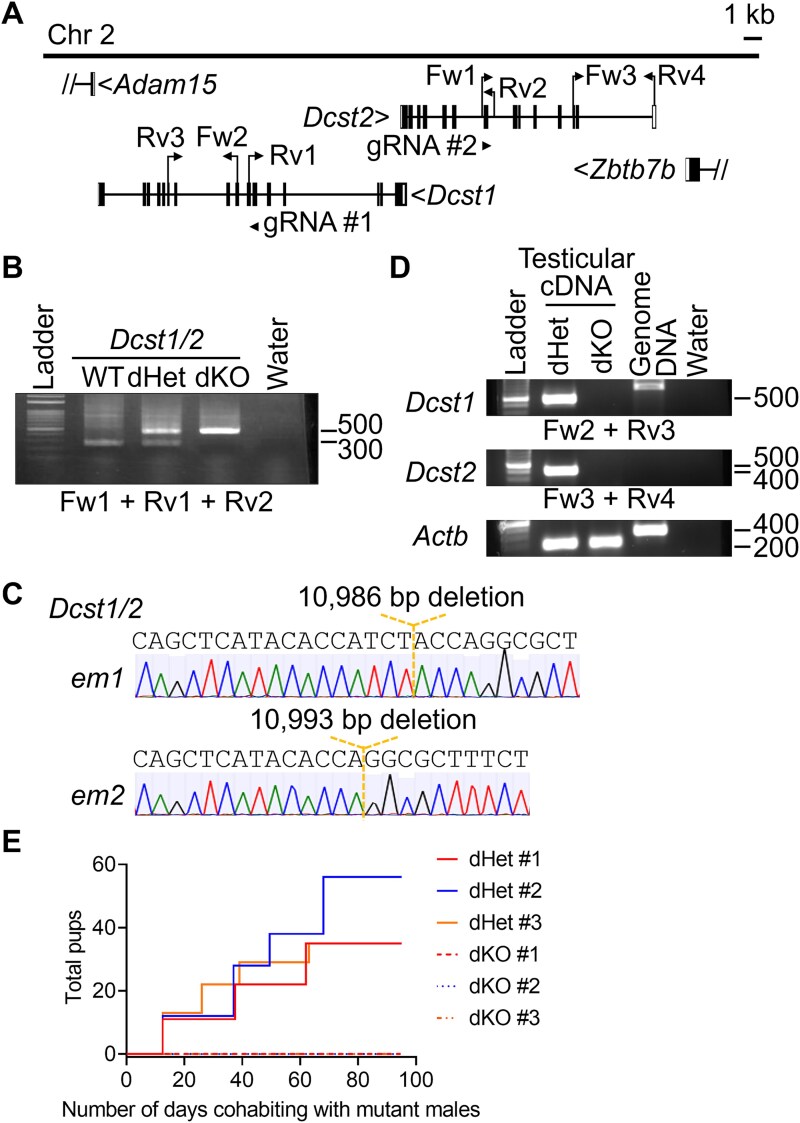
Male rats lacking *Dcst1* and *Dcst2* are infertile. (A) Guide RNA (gRNA) design. The genomic region encoding rat *Dcst1* and *Dcst2* (chr 2: 177,062,300–177,096,000) is shown. The signs (“<” and “>”) before and after the gene name indicate the transcriptional direction. Black boxes, arrows, and arrowheads indicate protein-coding regions, PCR primers, and gRNA, respectively. (B) Genotyping PCR. The primers shown in Panel A were used for genotyping. Water was used as a negative control. WT, wild-type; dHet, double heterozygous; dKO, double knockout. (C) DNA sequencing. The KO allele lacked 10,986 bases (chr 2: 177,071,539–177,082,524) or 10,993 bases (chr 2: 177,071,536–177,082,528), corresponding to exons 1–6 of *Dcst1* (ENSRNOT00000027986.10) and exons 1–7 of *Dcst2* (ENSRNOT00000081189.3). (D) Detection of rat *Dcst1* and *Dcst2* mRNAs. To examine the expression of *Dcst1* and *Dcst2* mRNAs in the testis of *Dcst1/2* dKO mice, exons 9–12 of *Dcst1* and exons 12–15 of *Dcst2*, which remained in the KO allele, were amplified. PCR with genomic DNA was used to examine the contamination of genomic DNA in testicular cDNA. Transcripts of *Dcst1* and *Dcst2* were not detected in KO testes. Actin, beta (*Actb*) was used as the loading control. Data reproducibility was verified using two technical replicates. (E) Male fertility. A mutant male was cohabited with two WT females for more than two months. Females cohabiting with *Dcst1/2* dKO males did not deliver pups, indicating that *Dcst1/2* dKO male rats were infertile.

To examine the physiological functions of rat *Dcst1* and *Dcst2*, we attempted to delete the entire coding region of these genes. However, *Adam15* and *Zbtb7b* (also known as *Th-POK*) are close to *Dcst1* and *Dcst2* (Figure 1A), and the disruption of mouse *Zbtb7b* reduces female fertility in genetically modified mice [24, 25]. The gRNAs were therefore designed in exon 6 of *Dcst1* and exon 7 of *Dcst2* to avoid influencing the expression levels of the neighboring genes (Figure 1A). A mixture of gRNA and Cas9 was injected into a pronucleus of fertilized eggs to obtain founder (F0) mutants lacking the genomic region encoding *Dcst1* and *Dcst2* (sixty transferred eggs, seven delivered pups, and four mutant pups). The F0 mutants were subsequently mated with WT rats to obtain the F1 mutants. Next, F1 × F1 intercrosses were used to establish dKO mutants lacking 10,986 or 10,993 nucleotides corresponding to the genomic region encoding exons 1–6 of rat *Dcst1* and exons 1–7 of rat *Dcst2* (Figure 1B and C). To examine the expression of *Dcst1* and *Dcst2* mRNAs, exons 9–12 of *Dcst1* and exons 12–15 of *Dcst2*, which remained in the KO allele, were amplified using reverse transcription PCR (RT-PCR). No bands were detected in dKO testes, indicating that there were no transcripts from the remaining genomic region of *Dcst1* and *Dcst2* (Figure 1D). When *Dcst1/2* WT and dKO females were caged with either *Dcst1/2* WT or dHet males, *Dcst1/2* dKO females mated with WT males delivered a similar number of pups as WT females [11.5 ± 3.6 pups/delivery (11 deliveries, WT females), 10.4 ± 3.4 pups/delivery (18 deliveries, dKO females)]. However, WT females caged with *Dcst1/2* dKO males did not deliver a pup [42.0 ± 12.1 total pups (dHet males), 0 pup (dKO males)] (Figure 1E and Supplementary Table S1), indicating that *Dcst1/2* dKO male rats were completely infertile.

### Spermatogenesis of *Dcst1/2* dKO rats was comparable to that of control rats

**Figure 2:**
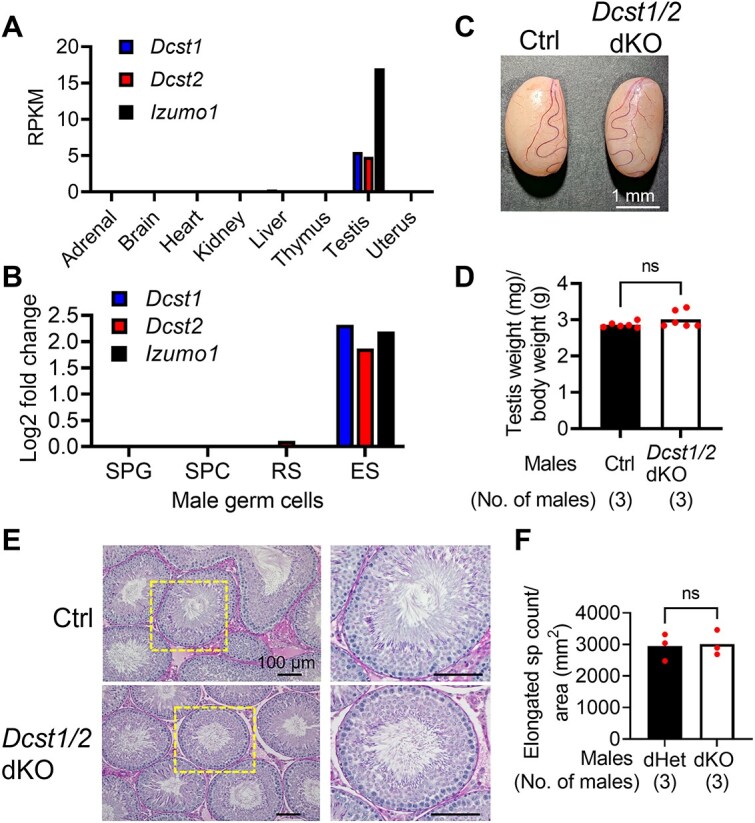
*Dcst1/2* dKO rats show normal spermatogenesis. (A) Expression of rat *Dcst1* and *Dcst2* in multiple tissues. Based on published RNA-seq data, the expression patterns of rat *Dcst1* and *Dcst2* in the multiple tissues were shown. Both genes were restrictedly expressed in the testis. Rat *Izumo1*, a gene required for sperm–egg binding, was used to compare expression patterns. (B) Expression of rat *Dcst1* and *Dcst2* in each developmental stage of male germ cells. Testicular germ cells expressing rat *Dcst1* and *Dcst2* were examined using a published single-cell RNA-seq database. Transcripts of both genes were abundant in elongating spermatids, corresponding to the expression pattern of rat *Izumo1*. SPG, spermatogonia; SPC, spermatocytes; RS, round spermatids; ES, elongating spermatids. (C) Gross morphology of a testis of *Dcst1/2* mutants*.* There was no marked difference between the testes of control (ctrl) and *Dcst1/2* dKO males. Data reproducibility was evaluated using three biological replicates. (D) Testis weight (mg)/body weight (g). The testis weights shown in panel C were measured, and there was no difference between the testes of control and *Dcst1/2* dKO males (Mann–Whitney test, *p* = 0.2). ns: not significant. (E) Histological analysis using periodic acid Schiff–hematoxylin staining. Seminiferous tubules in stages VII–VIII (dotted areas) were enlarged (right panels). There was no overt difference in spermatogenesis between the seminiferous tubules of control and *Dcst1/2* dKO mice. Data reproducibility was evaluated using three biological replicates. (F) Counts of elongated spermatids per the diameter of a seminiferous tubule. The elongated spermatids in the seminiferous tubules at stages VII–VIII (five seminiferous tubules per male) were counted. The number of elongated spermatids in *Dcst1/2* dKO testes was similar to that in control testes (Mann–Whitney test, *p* > 0.99). ns: not significant.

Based on the NCBI Gene Expression Omnibus database, rat *Dcst1* and *Dcst2* showed restricted expression in the testis (Figure 2A). Furthermore, *Dcst1* and *Dcst2* mRNAs were abundant in elongating spermatids, as determined using published single-cell RNA-seq data (Figure 2B). These results were consistent with the expression pattern of rat *Izumo1* mRNA. Spermatogenesis in *Dcst1/2* dKO testis was therefore examined to determine the cause of male infertility in *Dcst1/2* dKO mice. The gross morphology and weight of *Dcst1/2* dKO rat testes were similar to those of control rats {testis weight (mg)/body weight (g): 2.86 ± 0.08 [control (ctrl)], and 3.01 ± 0.23 (dKO)} (Figure 2C and D and Supplementary Table S2). Each developmental stage of male germ cells in the seminiferous tubules of *Dcst1/2* dKO testes was observed using histological analysis with periodic acid–Schiff (PAS)–hematoxylin staining. The dKO seminiferous tubules contained male germ cells at all developmental stages, similar to the control seminiferous tubules (Figure 2E). There was no marked difference in the number of elongated spermatids between control and *Dcst1/2* dKO seminiferous tubules (Figure 2F and Supplementary Table S3). These results indicate normal spermatogenesis in *Dcst1/2* dKO rats.

### *Dcst1/2* dKO sperm could bind to, but not fuse with, eggs

**Figure 3:**
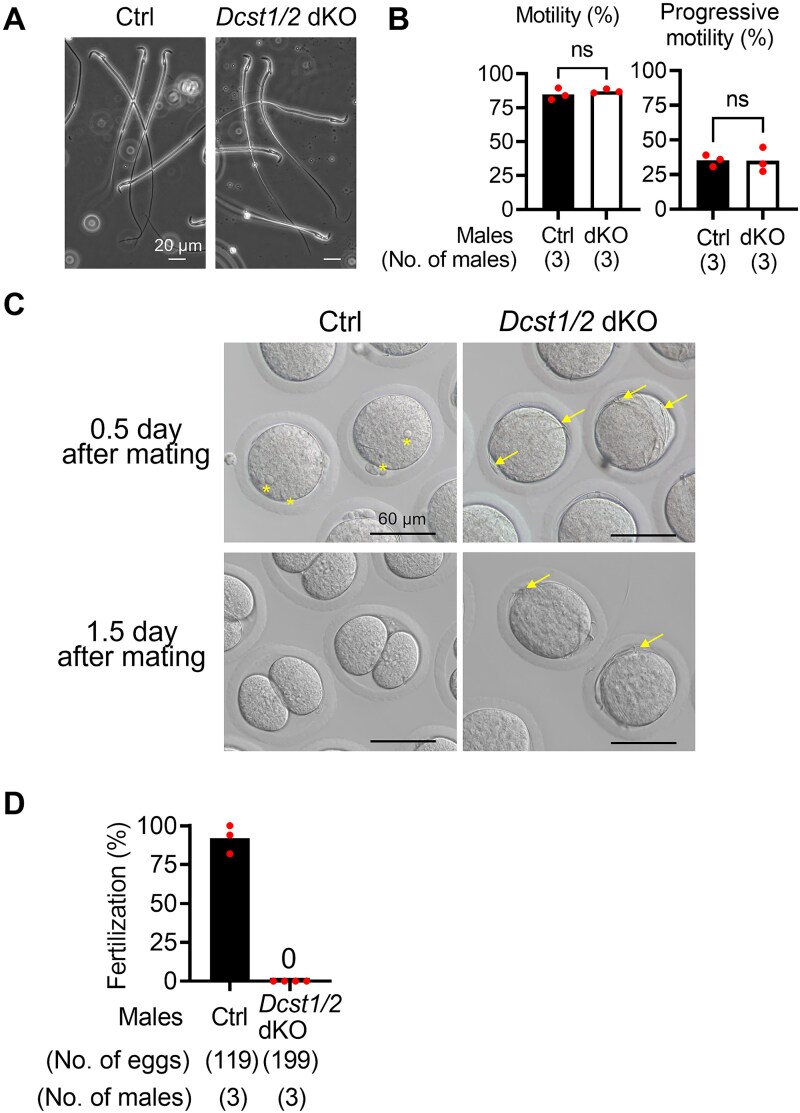
*Dcst1/2* dKO sperm accumulated in the perivitelline space of eggs collected from the females after mating. (A) Observation of sperm morphology. The morphology of *Dcst1/2* dKO sperm from the cauda epididymis was similar to that of control sperm. Data reproducibility was evaluated using three biological replicates. (B) Evaluation of sperm motility. Sperm motility after 3 h of incubation in mHTF medium was analyzed using Computer-Assisted System Analysis (CASA) software. There were no differences in motility and progressive motility between control and *Dcst1/2* dKO sperm [Mann–Whitney test, *p* = 0.70 (motility), and *p* > 0.99 (progressive motility)]. ns: not significant. (C) Eggs collected from females after mating. Eggs were collected from the ampulla of hormone-treated females mated with control or *Dcst1/2* dKO males. Embryos with pronuclei (PN, asterisks) and two-cell embryos were observed after 0.5 and 1.5 days of mating with the control males. However, embryos with pronuclei and two-cell embryos were not observed in females mated with *Dcst1/2* dKO males and *Dcst1/2* dKO sperm accumulated in the perivitelline space (arrows). Data reproducibility was verified using more than three biological replicates. (D) Fertilization rates after mating. The results shown in Panel C were quantified. No fertilized eggs (2PN embryos) were obtained from females mated with *Dcst1/2* dKO males.

**Figure 4:**
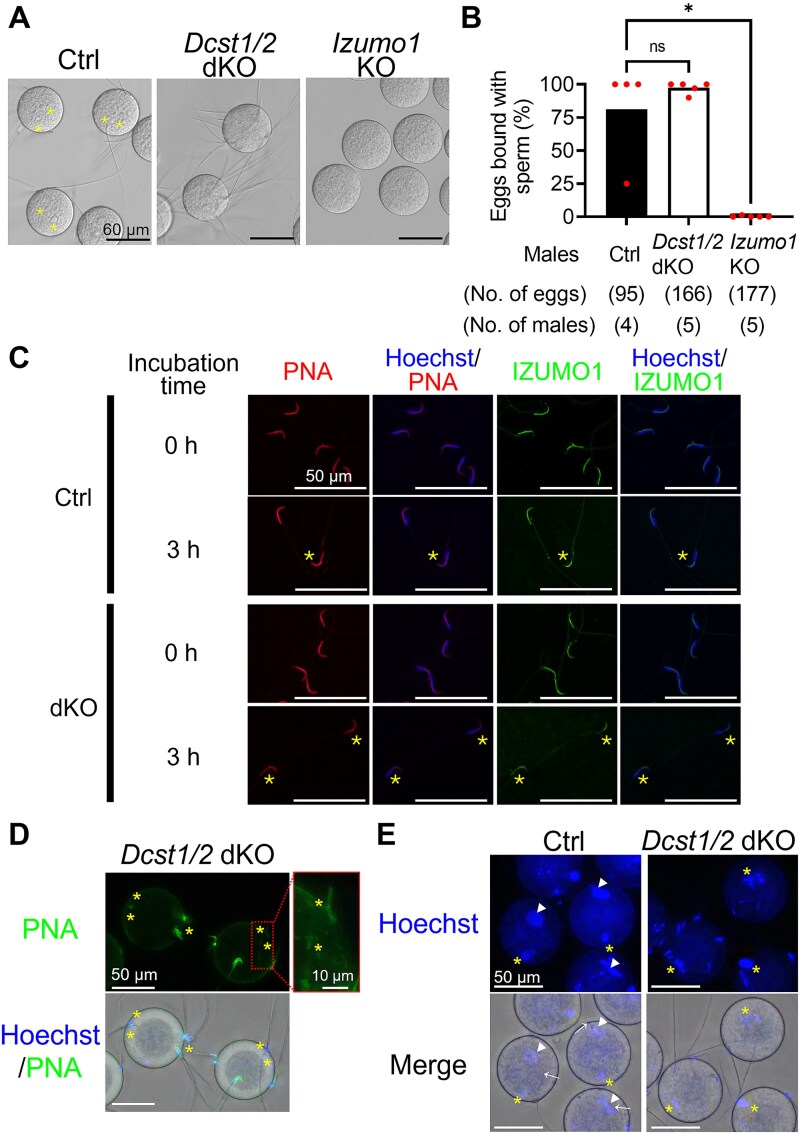
*Dcst1/2* dKO sperm can bind to but not fuse with eggs. (A) Evaluation of the binding and fusion of sperm and eggs. More than 4 h after insemination, the eggs were washed with mHTF medium. Control sperm bound to the oolemma and pronuclei (asterisks) were observed in these eggs. *Dcst1/2* dKO sperm could bind to the oolemma; however, no pronuclei were found. *Izumo1* KO sperm were used as the control to evaluate sperm–egg binding ability. Data reproducibility was verified using more than four biological replicates. (B) Percentages of eggs bound with sperm. The results shown in Panel A were quantified. There was no difference between ctrl and *Dcst1/2* dKO sperm [Kruskal–Wallis test, *p* > 0.99 (ctrl vs *Dcst1/2* dKO sperm), and *p* = 0.02 (ctrl vs *Izumo1* KO sperm)]. ^*^p < 0.05; ns, not significant. (C) Observation of sperm acrosome reaction. Cauda epididymal sperm were dispersed in PBS or incubated in mHTF drops for 3 h. After fixation with methanol, the sperm were stained with Alexa Fluor 568-conjugated lectin PNA and an anti-human IZUMO1 antibody. The specificity of the anti-human IZUMO1 antibody was examined using *Izumo1* KO rat sperm (see Supplementary Figure S1B). Sperm nuclei were counterstained with Hoechst33342. Asterisks indicate acrosome-reacted sperm. Data reproducibility was verified using four biological replicates. (D) Observation of the acrosomal status of sperm binding with the oolemma. Sperm pre-incubated in mHTF medium were stained with Alexa Fluor 488-conjugated lectin PNA for 1 h (total incubation time: 7 h), and then they were inseminated into ZP-free eggs. One to two hours after insemination, the acrosomal status of the sperm binding with the oolemma was observed. The PNA signals of some *Dcst1/2* dKO sperm binding with the oolemma became faint (^*^), indicating that these sperm were during and after the acrosome reaction (see Supplementary Figure S3). This result indicates that the acrosome-reacted sperm of *Dcst1/2* dKO rats could bind to the oolemma. The red-dotted area was enlarged (right panel). The brightness was changed in the enlarged image. The data reproducibility was evaluated using three biological replicates. (E) Observation of sperm nuclei bound to the oolemma. To examine whether the nuclei of sperm bound to the oolemma swelled, eggs were stained with Hoechst33342. Swollen sperm nuclei were found only when the sperm had fused with the oolemma. Swollen nuclei (arrowheads) and flagella (arrows) of the control sperm were observed, but those of the *Dcst1/2* dKO sperm were not. This result indicates that *Dcst1/2* dKO sperm can bind to, but not fuse with, the oolemma. Asterisks indicate the oocyte chromosomes. Data reproducibility was verified using three biological replicates.

Since spermatogenesis in *Dcst1/2* dKO rats was normal, the fertilization abilities of these sperm were determined. No overt abnormalities were observed in the morphology and motility of *Dcst1/2* dKO sperm [motility: 84.8 ± 4.3 (ctrl) and 87.2 ± 1.5 (dKO); progressive motility: 35.2 ± 4.2 (ctrl) and 34.9 ± 8.9 (dKO)] (Figure 3A–B and Supplementary Table S4). Eggs were collected from females mated with either control or *Dcst1/2* dKO males to observe sperm–egg interactions. Although fertilized eggs with pronuclei (2PN) and two-cell embryos were found in females mated with control males, no fertilized eggs were detected in females mated with *Dcst1/2* dKO males [fertilization rates (percentage of eggs with 2PN): 92.3 ± 9.0% (dHet) and 0% (dKO)] (Figure 3C and D and Supplementary Table S5). *Dcst1/2* dKO sperm accumulated in the perivitelline spaces of eggs (Figure 3C). This result suggests that *Dcst1/2* dKO sperm can pass through the ZP, but do not interact with the oolemma. These sperm were then incubated with ZP-removed eggs to examine their binding and fusion abilities. The control sperm could bind to and fuse with the oolemma (Figure 4A). Although *Dcst1/2* dKO sperm could bind to the oolemma, pronuclear formation was not observed (Figure 4A). Moreover, *Izumo1* KO sperm did not bind to the oolemma (Figure 4A), which is consistent with a previous study [5]. The population of eggs bound with *Dcst1/2* dKO sperm was comparable to that of eggs bound with control sperm [eggs bound to sperm: 81.3 ± 37.5% (ctrl), 97.4 ± 4.3% (*Dcst1/2* dKO), and 0.3 ± 0.6% (*Izumo1* KO)] (Figure 4B and Supplementary Table S6). Immunostaining was used to determine whether IZUMO1, a sperm protein required for sperm–egg binding, existed in rat *Dcst1/2* dKO sperm. Unfortunately, when live sperm were stained, fluorescence signals were observed even in *Izumo1* KO sperm (Supplementary Figure S1A). When fresh sperm fixed with methanol were stained, fluorescence signals were detected in control and *Dcst1/2* dKO sperm (Figure 4C) but not in *Izumo1* KO sperm (Supplementary Figure S1B). This result indicates that IZUMO1 remains in *Dcst1/2* dKO sperm and that these dKO sperm can bind to the oolemma through the remaining IZUMO1. The immunostaining results indicated that the anti-human IZUMO1 antibody worked well for fixed sperm, but not for live sperm, at least under the conditions of the protocols in the current study.

**Figure 5:**
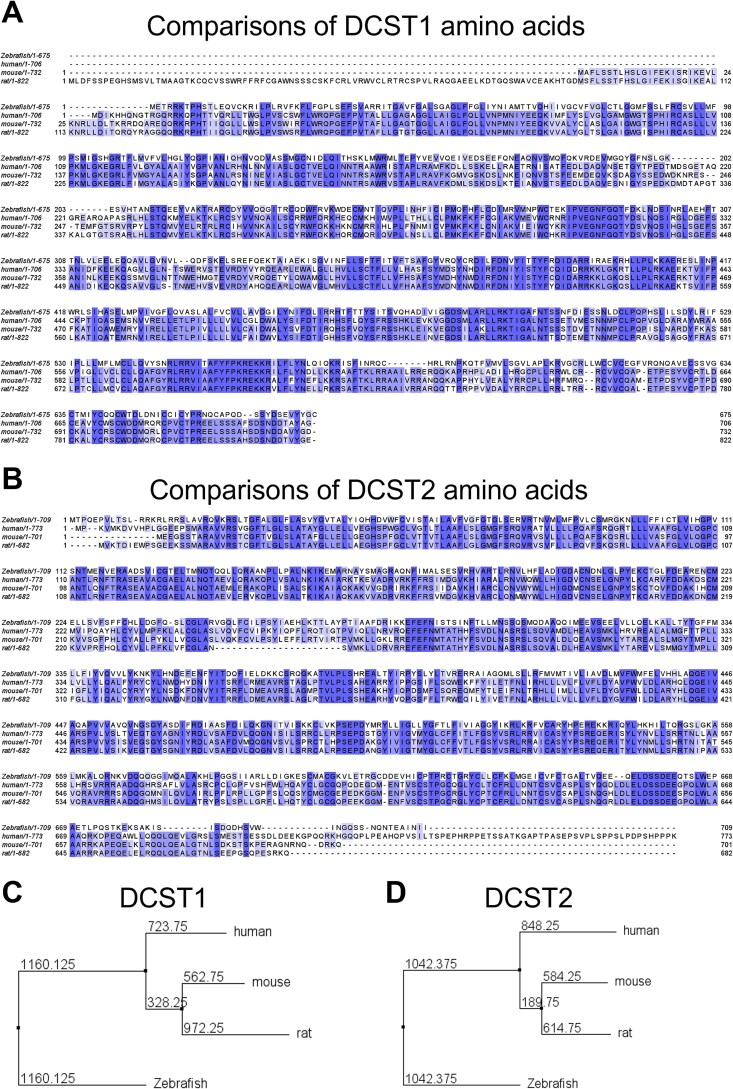
Comparisons of amino acid sequences of DCST1 and DCST2 in zebrafish, mice, rats, and humans, and phylogenetic analysis. (A) Comparisons of DCST1 amino acid sequences. Characters highlighted in dark or light blue indicate amino acids conserved among all or some species, respectively. (B) Comparison of DCST2 amino acid sequences. (C) Phylogenetic analysis using zebrafish, mouse, rat, and human DCST1 amino acid sequences. The values for each branch indicate the branch length, which shows genetic changes. (D) Phylogenetic analysis using zebrafish, mouse, rat, and human DCST2 amino acid sequences.

Although only acrosome-reacted sperm interact with the oolemma under natural conditions, mouse acrosome-intact sperm artificially bind to the oolemma when these sperm are inseminated with ZP-removed eggs [5]. Therefore, whether *Dcst1/2* dKO sperm underwent an acrosome reaction was determined. To compare the acrosome reactions, control and *Dcst1/2* dKO sperm were dispersed in PBS (incubation time: 0 h) or incubated in the mHTF medium for 3 h, and the sperm after fixation were stained with PNA and an anti-IZUMO1 antibody. Because PNA binds mainly to the outer acrosomal membrane [26], PNA signals become faint after the acrosome reaction. IZUMO1 is localized in the inner and outer acrosomal membranes of the acrosome-intact sperm, and then moves to the entire head by the translocation of IZUMO1 from the outer acrosomal membrane to the sperm plasma membrane after the acrosome reaction [27]. Before incubation, the fluorescence signals of PNA and IZUMO1 were detected mainly in the acrosome cap of the sperm, indicating that these sperm were before the acrosome reaction (Figure 4C). As the incubation time increased, the signals became faint or spread to the head (Figure 4C), indicating that these sperm were during and after the acrosome reaction. The distributions of PNA and IZUMO1 were consistent with those of PNA and IZUMO1 in sperm treated with the Ca^2+^ ionophore, A23187, to artificially induce the acrosome reaction (Supplementary Figure S1C). There was no marked difference in the population of acrosome-reacted sperm between control and *Dcst1/2* dKO rats (Supplementary Figure S2, and Supplementary Tables S7 and S8), indicating that *Dcst1/2* dKO sperm underwent a normal acrosome reaction, similar to control sperm.

To determine whether rat *Dcst1/2* dKO sperm bound to the oolemma underwent an acrosome reaction, live-cell staining using a PNA lectin was conducted. As described above, PNA binds mainly to the outer acrosomal membrane, and the signals become faint after the acrosome reaction [26]. PNA signals were detected at the acrosome cap of most live sperm incubated in mHTF medium for 2 h (Supplementary Figure S3), corresponding to the acrosome-intact sperm (Figure 4C). After 7-h incubation of sperm, the PNA signals became faint (Supplementary Figure S3), consistent with acrosome-reacted sperm (Figure 4C). As time-dependent changes in PNA signals were detected, the acrosomal status of *Dcst1/2* dKO sperm bound to the oolemma was evaluated. When *Dcst1/2* dKO rat sperm pre-stained with PNA lectin were inseminated into ZP-free eggs, acrosome-reacted sperm with faint PNA signals could bind to the oolemma (Figure 4D). To determine whether *Dcst1/2* dKO sperm fused with the oolemma, the staining of the sperm nucleus with Hoechst33342 was conducted, as only the fused sperm nuclei would swell in the egg. Five hours of insemination, most control sperm bound to eggs had at least one swollen sperm nucleus [eggs with swollen nuclear/total eggs (*n* = 3): 53/63]. In contrast, none of the *Dcst1/2* dKO sperm bound to eggs had swollen sperm nuclei [eggs with swollen nuclear/total eggs (*n* = 3): 0/46] (Figure 4E and Supplementary Table S9). Therefore, the acrosome-reacted sperm of *Dcst1/2* dKO rats could bind to, but not fuse with, the oolemma, leading to male infertility.

Unlike zebrafish Dcst1/2, rat DCST1/2 is required for sperm–egg fusion. This is consistent with the function of mouse DCST1/2. When amino acid sequences in mouse, rat, and zebrafish DCST1/2 were compared, the homology rates of rat DCST1/2 against mouse DCST1/2 were higher than those of zebrafish Dcst1/2 (Figure 5A and B) [DCST1: 81.7% (rat vs. mouse), 74.5% (rat vs. human), and 39.0% (rat vs. zebrafish); DCST2: 83.2% (rat vs. mouse), 77.9% (rat vs. human), and 37.7% (rat vs. zebrafish)]. Phylogenetic analysis showed that human DCST1/2 was closer to mouse and rat DCST1/2 than to zebrafish Dcst1/2 (Figure 5C and D). Thus, we theorized that the roles of mouse and rat DCST1/2 in sperm–egg fusion were conserved among many mammals, including humans.

## Supplementary Material

Figure_S1_Nakano_et_al_ioag111

Figure_S2_Nakano_et_al_ioag111

Figure_S3_Nakano_et_al_ioag111

Supplementary_Tables_Nakano_et_al_20260424_ioag111(1)

Supplementary_Figures_Nakano_et_al_20260424_ioag111

## Data Availability

The data underlying this article will be shared upon reasonable request from the corresponding authors. Masahito Ikawa holds the position of Associate Editor for Biology of Reproduction and has not peer reviewed or made any editorial decisions for this paper.
