# Supplementary material for: Rat DCST1 and DCST2 are essential for sperm–egg fusion
Source: Biol Reprod. 2026 May 23;115(2):539–49. doi: 10.1093/biolre/ioag111 (PMC13482280; doi:10.1093/biolre/ioag111)
Supplement: Supplementary file 1 [file supplementary_figures_nakano_et_al_20260424_ioag111.pdf]

Supplementary Figure S1

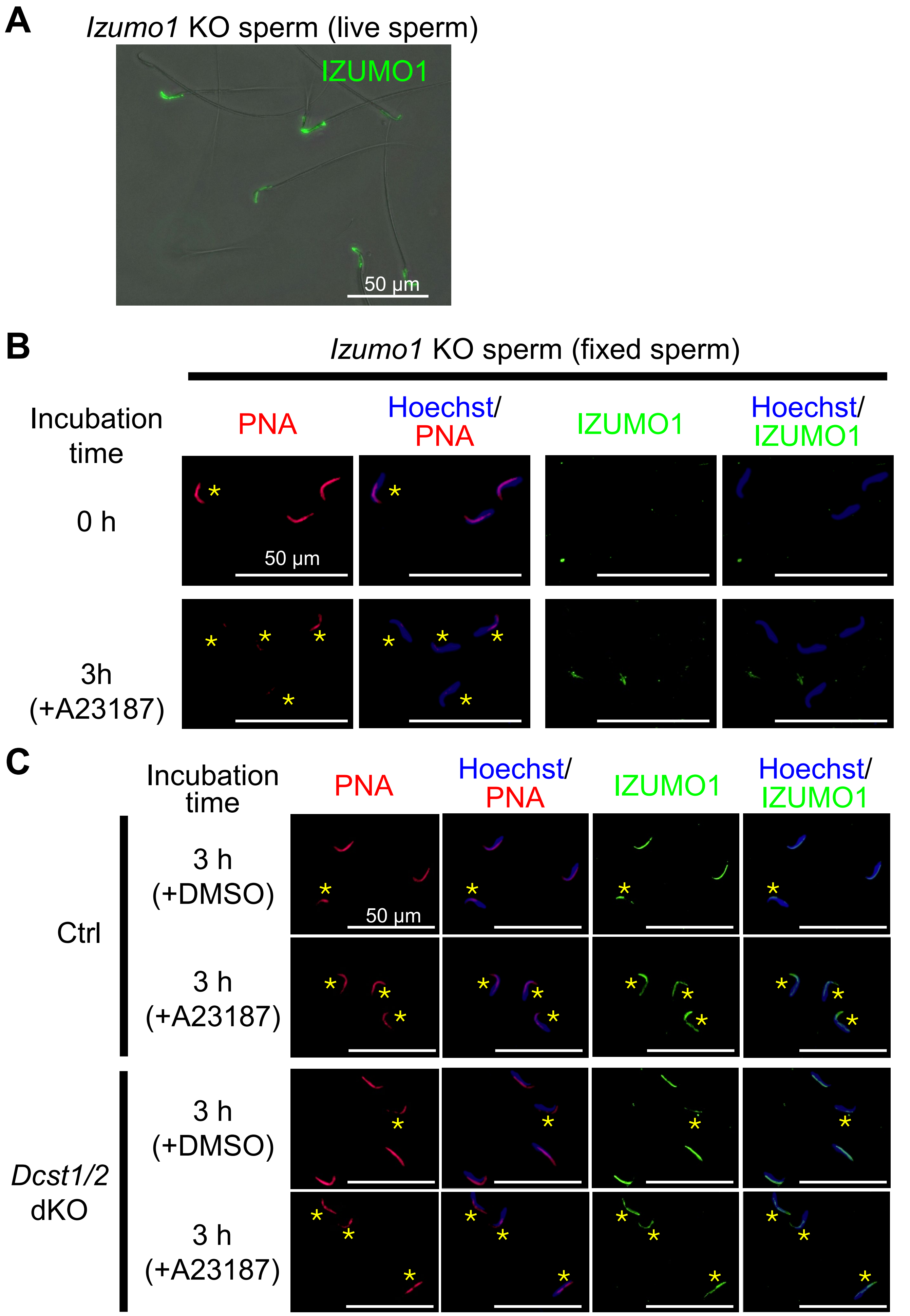

Supplementary Figure S1. Specificity of an IZUMO1 antibody and the observation of sperm acrosome reaction using PNA lectin and an IZUMO1 antibody.

A) Immunostaining using live sperm. *Izumo1* KO sperm pre-incubated in mHTF medium were incubated with an anti-human IZUMO1 antibody for 30 minutes, followed by the incubation of a secondary antibody (goat anti-rabbit IgG Alexa Fluor 488) for 30 minutes. Some non-specific signals were observed in the live sperm of *Izumo1* KO rats. The data reproducibility was verified using two biological replicates.

B) Immunostaining using fixed sperm. *Izumo1* KO rat sperm were dispersed in PBS or incubated in mHTF medium with A23187, a calcium ionophore. After washing with PBS, sperm were smeared on a glass slide, fixed with methanol, and then were used for immunostaining. PNA lectin was used to observe the acrosomal status. No fluorescence signals of IZUMO1 were found in acrosome-intact and acrosome-reacted sperm (asterisks) of *Izumo1* KO rats. The data reproducibility was verified using three biological replicates.

C) Observation of sperm acrosome reaction. To induce the acrosome reaction, some sperm pre-incubated in mHTF medium were treated with calcium ionophore A23187 (total incubation time: 3 hours). DMSO-treated sperm were used as the control. After fixation with methanol, these sperm were stained with Alexa Fluor 568-conjugated lectin PNA and an anti-human IZUMO1 antibody. Sperm nuclei were counterstained with Hoechst33342. Asterisks show the acrosome-reacted sperm. The data reproducibility was verified using three biological replicates.

Supplementary Figure S2

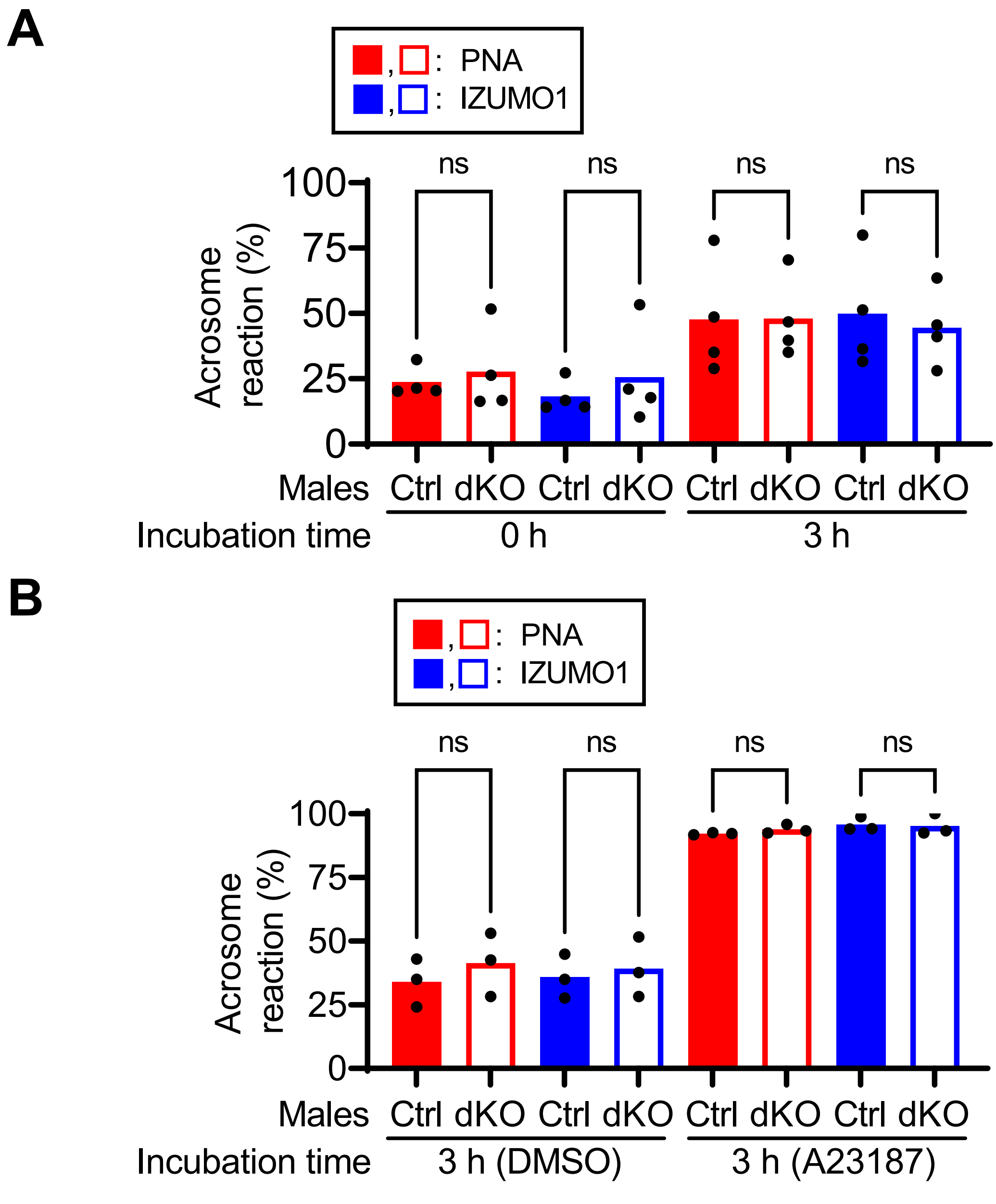

Supplementary Figure S2.*Dcst1/2* dKO sperm underwent an acrosome reaction, at a similar level to control sperm.
A) Population of acrosome reactions of sperm pre-incubated in mHTF medium. The acrosome-reacted sperm in Figure 4C were counted. There was no difference in the population of acrosome reactions between control and *Dcst1/2* dKO sperm [Kruskal–Wallis test, 0 hour: > 0.99 (PNA) and > 0.99 (IZUMO1), and 3 hours: > 0.99 (PNA) and > 0.99 (IZUMO1)]. The data reproducibility was verified using four biological replicates. ns, not significant.
B) Population of acrosome reactions of A23187-treated sperm. The acrosome-reacted sperm in supplementary Figure S1C were counted. There was no difference in the population of acrosome reactions between the control and *Dcst1/2* dKO sperm [Kruskal–Wallis test, DMSO: > 0.99 (PNA) and > 0.99 (IZUMO1), and A23187: > 0.99 (PNA) and > 0.99 (IZUMO1)]. The data reproducibility was verified using three biological replicates. ns, not significant.

Supplementary Figure S3

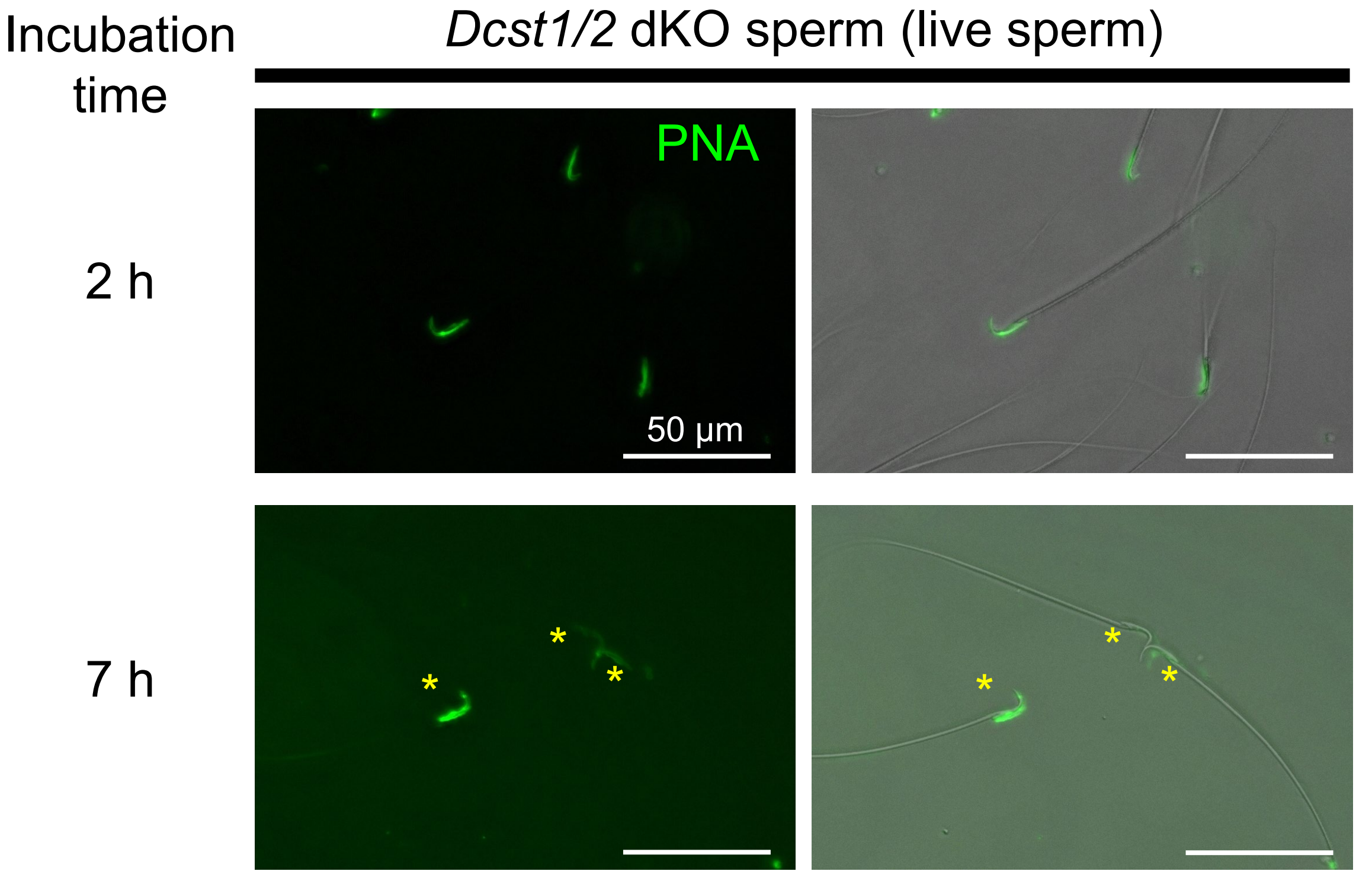

Supplementary Figure S3. Observation of the acrosomal status in live sperm using PNA lectin. Sperm pre-incubated in mHTF medium were treated with Alexa Fluor 488-conjugated lectin PNA for 1 hour (total incubation time: 2 or 7 hours), and then the PNA signal was observed under a fluorescence microscope. Asterisks (*) show the acrosome-reacted sperm. The data reproducibility was verified using three biological replicates.
