# Supplementary material for: Rat DCST1 and DCST2 are essential for sperm–egg fusion
Source: Biol Reprod. 2026 May 23;115(2):539–49. doi: 10.1093/biolre/ioag111 (PMC13482280; doi:10.1093/biolre/ioag111)
Supplement: Supplementary file 2 [file supplementary_tables_nakano_et_al_20260424_ioag111(1).pdf]

Supplementary Table S1. Male fertility of *Dcst1/2* dHet and dKO rats

Male #	Genotype	Mating start	Mating stop	DOB (upper)/ Delivered pups (lower)			
1	*em1/wt*	7th Oct 2024	10th Jan 2025	1st Nov 2024<br>11	26th Nov 2024<br>11	20th Dec 2024<br>13	
2	*em1/wt*	7th Oct 2024	10th Jan 2025	1st Nov 2024<br>12	25th Nov 2024<br>16	26th Nov 2024<br>10	1st Jan 2025<br>18
3	*em1/wt*	7th Oct 2024	10th Jan 2025	1st Nov 2024<br>13	3rd Nov 2024<br>9	27th Nov 2024<br>7	21st Dec 2024<br>6
4	*em1/em1*	7th Oct 2024	10th Jan 2025	No pup			
5	*em1/em1*	7th Oct 2024	10th Jan 2025	No pup			
6	*em1/em2*	7th Oct 2024	10th Jan 2025	No pup			

To check male fertility, *Dcst1/2* dHet and dKO males were cohabited with two WT females. Females cohabiting with *Dcst1/2* dKO males did not deliver pups (also see Figure 1E). *em*: enzyme mutation.

Supplementary Table S2. Testis weight (mg)/body weight (g)

Male #	dHet	dKO
1	2.78	2.84
	2.83	2.84
2	3.00	2.84
	2.89	2.92
3	2.85	3.34
	2.80	3.26

Supplementary Table S3. Counts of elongated sperm in each seminiferous tubule

Males	Seminiferous tubule #	No. of elongated sperm	Area of the seminiferous tubules (mm^2)	Elongated sperm count/area (mm^2)
dHet #1	1	192	0.073	2630.14
	2	210	0.058	3620.69
	3	247	0.068	3632.35
	4	218	0.061	3573.77
	5	147	0.047	3127.66
dHet #2	1	143	0.061	2344.26
	2	153	0.061	2508.20
	3	163	0.060	2716.67
	4	144	0.064	2250.00
	5	126	0.049	2571.43
dHet #3	1	166	0.046	3608.70
	2	171	0.051	3352.94
	3	190	0.070	2714.29
	4	346	0.122	2836.07
	5	162	0.060	2700.00
dKO #1	1	257	0.086	2988.37
	2	222	0.064	3468.75
	3	170	0.055	3090.91
	4	158	0.066	2393.94
	5	144	0.057	2526.32
dKO #2	1	183	0.066	2772.73
	2	187	0.051	3666.67
	3	163	0.052	3134.62
	4	182	0.045	4044.44
	5	177	0.048	3687.50
dKO #3	1	281	0.083	3385.54
	2	283	0.103	2747.57
	3	207	0.084	2464.29
	4	211	0.080	2637.50
	5	194	0.088	2204.55

Supplementary Table S4. Evaluation of sperm parameters using CASA

Experimental #	Motility (%)		Progressive motility (%)	
	dHet	dKO	dHet	dKO
1	81.0	85.9	30.7	32.7
2	89.5	86.8	39.1	27.3
3	84.0	88.9	35.9	44.7

Supplementary Table S5. Fertilization rates obtained by mating with control and *Dcst1/2* dKO males

Experimental #	Ctrl (2 PN eggs/total eggs)	dKO (2 PN eggs/total eggs)
1	42/51 (82.4%)	0/85 (0%)
2	34/36 (94.4%)	0/30 (0%)
3	32/32 (100%)	0/56 (0%)
4		0/28 (0%)

Supplementary Table S6. Percentages of eggs bound with sperm

Experimental #	Ctrl	*Dcst1/2* dKO	*Izumo1* KO
1	35/35 (100%)	38/38 (100%)	0/37 (0%)
2	20/20 (100%)	66/68 (97.1%)	1/80 (1.3%)
3	5/20 (25.0%)	20/20 (100%)	0/20 (0%)
4	20/20 (100%)	20/20 (100%)	0/20 (0%)
5		18/20 (90.0%)	0/20 (0%)

Supplementary Table S7. Percentages of sperm acrosome reaction

Experimental #	PNA				IZUMO1			
	0 h (PBS) (AR sperm/total sperm)		3 h (mHTF) (AR sperm/total sperm)		0 h (PBS) (AR sperm/total sperm)		3 h (mHTF) (AR sperm/total sperm)	
	Ctrl	dKO	Ctrl	dKO	Ctrl	dKO	Ctrl	dKO
1	13/64 (20.3%)	8/48 (16.7%)	26/74 (35.1%)	37/79 (46.8%)	9/64 (14.1%)	5/48 (10.4%)	27/74 (36.5%)	36/79 (45.6%)
2	32/99 (32.3%)	31/60 (51.7%)	34/70 (48.6%)	29/73 (39.7%)	27/99 (27.3%)	32/60 (53.3%)	36/70 (51.4%)	30/73 (41.1%)
3	16/78 (20.5%)	12/73 (16.4%)	11/38 (28.9%)	20/57 (35.1%)	13/78 (16.7%)	13/73 (17.8%)	12/38 (31.6%)	16/57 (28.1%)
4	9/42 (21.4%)	10/38 (26.3%)	39/50 (78.0%)	31/44 (70.5%)	6/42 (14.3%)	8/38 (21.1%)	40/50 (80.0%)	28/44 (63.6%)

Supplementary Table S8. Percentages of sperm acrosome reaction

Experimental #	PNA				IZUMO1			
	+ DMSO (AR sperm/total sperm)		+ A23187 (AR sperm/total sperm)		+ DMSO (AR sperm/total sperm)		+ A23187 (AR sperm/total sperm)	
	Ctrl	dKO	Ctrl	dKO	Ctrl	dKO	Ctrl	dKO
1	21/60 (35.0%)	26/61 (42.6%)	77/84 (91.7%)	56/60 (93.3%)	21/60 (35.0%)	23/61 (37.7%)	79/84 (94.0%)	56/60 (93.3%)
2	21/49 (42.9%)	34/64 (53.1%)	87/94 (92.6%)	46/48 (95.8%)	22/49 (44.9%)	33/64 (51.6%)	93/94 (98.9%)	48/48 (100%)
3	14/58 (24.1%)	15/53 (28.3%)	47/51 (92.2%)	86/93 (92.5%)	16/58 (27.6%)	15/53 (28.3%)	48/51 (94.1%)	86/93 (92.5%)

Supplementary Table S9. Percentages of eggs with swollen sperm nuclei

Experimental #	Ctrl	*Dcst1/2* dKO
1	19/23 (83%)	0/22 (0%)
2	13/19 (68%)	0/10 (0%)
3	21/21 (100%)	0/14 (0%)
